# Draft Genome Sequence of the Polychlorinated Biphenyl-Degrading Bacterium Pseudomonas putida KF703 (NBRC 110666) Isolated from Biphenyl-Contaminated Soil

**DOI:** 10.1128/genomeA.00142-15

**Published:** 2015-03-19

**Authors:** Hikaru Suenaga, Atsushi Yamazoe, Akira Hosoyama, Nobutada Kimura, Jun Hirose, Takahito Watanabe, Hidehiko Fujihara, Taiki Futagami, Masatoshi Goto, Kensuke Furukawa

**Affiliations:** a Bioproduction Research Institute, National Institute of Advanced Industrial Science and Technology (AIST), Tsukuba, Japan; b Biological Resource Center, National Institute of Technology and Evaluation (NITE), Tokyo, Japan; c Faculty of Engineering, Department of Applied Chemistry, University of Miyazaki, Miyazaki, Japan; d Research Institute for Sustainable Humanosphere, Kyoto University, Kyoto, Japan; e Department of Food and Nutrition, Beppu University, Beppu, Japan; f Faculty of Agriculture, Education and Research Center for Fermentation Studies, Kagoshima University, Kagoshima, Japan; g Faculty of Agriculture, Laboratory of Future Creation Microbiology, Kyushu University, Fukuoka, Japan

## Abstract

Pseudomonas putida KF703 (NBRC 110666) utilizes biphenyl as a sole source of carbon and degrades polychlorinated biphenyls (PCBs). Here, we report the draft genome sequence of the KF703 strain, which provides insight into the molecular mechanisms of adaptation to an environment polluted by aromatic compounds.

## GENOME ANNOUNCEMENT

Polychlorinated biphenyls (PCBs) have been widely used for a variety of industrial purposes, and have become serious environmental contaminants. We have isolated 14 PCB-degrading bacterial strains (KF strains), including Pseudomonas putida KF703 (formerly known as Pseudomonas fluorescens KF703), from the soil near a biphenyl manufacturing plant in Kitakyushu, Japan by enrichment culture with biphenyl as a sole source of carbon (1). These KF strains belong to phylogenetically distinct genera and exhibit specific growth characteristics on various biphenyl derivatives. One of these strains, Pseudomonas pseudoalcaligenes KF707, was used to clone a *bph* gene cluster involved in biphenyl/PCB degradation for the first time (2, 3). We also revealed that an approximately 90-kb DNA region containing both the *bph* and salicylate catabolic *sal* genes (termed the *bph-sal* element) in the P. putida KF715 strain can be frequently transferred by conjugation to various P. putida strains (4, 5). Therefore, these KF strains are a suitable model for investigating molecular mechanisms in the adaptive evolution of xenobiotic-degrading bacteria in the natural environment. Here, we present the genomic features of the KF703 strain.

The draft genome sequence was determined by the National Institute of Technology and Evaluation (NITE) using 454 GS FLX + (Roche) and HiSeq 1000 (Illumina) systems. A standard fragment library was constructed for 454 sequencing, and 81,765 reads (57,267,221 bases) were obtained, while the pair-end sequencing with Illumina generated 5,916,170 reads (575,427,666 bases). The reads obtained by the two systems were assembled using the Newbler software package version 2.6 (Roche). The assembled genome is composed of 135 contigs (>500 bp) totaling 6,434,897 bases, with a G+C content of 62.1%. The *N*_50_ contig size and the largest contig size were 106,761 bp and 314,503 bp, respectively.

The draft genome sequence of the KF703 strain annotated using the RAST (Rapid Annotation using Subsystem Technology) server (6) contains 5,792 predicted coding DNA sequences (CDSs), four rRNAs (two, one, and one of 5S, 16S, and 23S), and 62 tRNA sequences. This RAST-based annotation revealed the presence of a large number of genes (*n* = 234) involved in the metabolism of aromatic compounds, such as biphenyl degradation (*n* = 23), benzoate degradation (*n* = 22), the catechol *ortho*-cleavage pathway (*n* = 12), and salicylate and gentisate catabolism (*n* = 16). The *bph* gene cluster (*bphRA1A2A3A4BCX0X1X2X3D*) is found in a single contig and is nearly identical to that of the KF707 strain in terms of gene organization and the amino acid sequence of the corresponding enzymes. In addition, the *bph-sal* element previously identified in the KF715 strain (5) was also found in the draft genome of the KF703 strain. These facts suggest that the *bph-sal* element was frequently spread by horizontal transfer between the KF strains in the natural environment.

### Nucleotide sequence accession numbers.

The draft genome sequence has been deposited at DDBJ/EMBL/GenBank under the accession numbers BBQL01000001 to BBQL01000135.
